# Analyzing Longitudinal Health Screening Data with Feature Ensemble and Machine Learning Techniques: Investigating Diagnostic Risk Factors of Metabolic Syndrome for Chronic Kidney Disease Stages 3a to 3b

**DOI:** 10.3390/diagnostics14080825

**Published:** 2024-04-17

**Authors:** Ming-Shu Chen, Tzu-Chi Liu, Mao-Jhen Jhou, Chih-Te Yang, Chi-Jie Lu

**Affiliations:** 1 Department of Healthcare Administration, College of Healthcare & Management, Asia Eastern University of Science and Technology, New Taipei City 220, Taiwan; 2 Graduate Institute of Business Administration, Fu Jen Catholic University, New Taipei City 242, Taiwan; 3 Department of Business Administration, Tamkang University, New Taipei City 251, Taiwan; 4 Artificial Intelligence Development Center, Fu Jen Catholic University, New Taipei City 242, Taiwan; 5 Department of Information Management, Fu Jen Catholic University, New Taipei City 242, Taiwan

**Keywords:** chronic kidney disease, metabolic syndrome, feature ensemble, machine learning, longitudinal data, health screening

## Abstract

Longitudinal data, while often limited, contain valuable insights into features impacting clinical outcomes. To predict the progression of chronic kidney disease (CKD) in patients with metabolic syndrome, particularly those transitioning from stage 3a to 3b, where data are scarce, utilizing feature ensemble techniques can be advantageous. It can effectively identify crucial risk factors, influencing CKD progression, thereby enhancing model performance. Machine learning (ML) methods have gained popularity due to their ability to perform feature selection and handle complex feature interactions more effectively than traditional approaches. However, different ML methods yield varying feature importance information. This study proposes a multiphase hybrid risk factor evaluation scheme to consider the diverse feature information generated by ML methods. The scheme incorporates variable ensemble rules (VERs) to combine feature importance information, thereby aiding in the identification of important features influencing CKD progression and supporting clinical decision making. In the proposed scheme, we employ six ML models—Lasso, RF, MARS, LightGBM, XGBoost, and CatBoost—each renowned for its distinct feature selection mechanisms and widespread usage in clinical studies. By implementing our proposed scheme, thirteen features affecting CKD progression are identified, and a promising AUC score of 0.883 can be achieved when constructing a model with them.

## 1. Introduction 

A sub-health condition (SHC) or sub-optimal health status refers to a condition characterized by decreased vitality, physiological function, and capacity for adaptation. However, it is yet to be medically diagnosed as a disease or functional somatic syndrome [1]. It is imperative to consider all SHC indicators to prevent chronic diseases and achieve better health outcomes. Metabolic syndrome (MetS) is a collection of indicators that define SHC risk and can assist in formulating strategies for preventing disease progression [2,3]. Metabolic factors, such as being overweight or obese and having hypertension, hyperlipidemia, and hyperglycemia, are critical metabolic changes that can increase the risk of chronic illness [4]. MetS increases the risk of developing various chronic diseases, such as a 2.5-fold higher risk of chronic kidney disease (CKD) [5], a 2.5-fold higher risk of myocardial infarction [4], a 2–4-fold higher risk of cardiovascular stroke, and a 5-fold higher risk of type II diabetes mellitus [6,7].

CKD is characterized by abnormal kidney function and is stratified into stages 1, 2, 3a, 3b, 4, and 5 according to the Kidney Disease Improving Global Outcomes’ (KDIGO) guideline [8]. Kidney diseases have become a major public health issue as they affect around 850 million individuals worldwide [9,10]. Patients with CKD often develop complications and MetS, accelerating their renal function deterioration, shortening the kidney lifespan, and ultimately increasing the incidence of CKD and intensifying its progression [11,12]. Both MetS and CKD are important risk factors for diverse complications [13,14]. Studies have demonstrated a positive correlation between MetS and CKD [15,16], and a MetS diagnosis effectively predicts CKD risk [16,17]. Among the existing studies, conventional statistical method usage is the approach that is commonly taken. 

Machine learning (ML) approaches, being relatively unaffected by the limitations of conventional statistical methods that rely on predefined assumptions and hypotheses, have found widespread use in detecting and predicting diseases at various stages, demonstrating promising performance [18,19]. ML approaches can proficiently analyze latent and intricate relationships and information that underlie multiple predictor variables/risk factors and outcomes [20]. Based on the feature selection results obtained from ML methods, the employment of the variable ensemble rule (VER) can aid in assessing the predictor variables of models to improve analytical outcomes. The VER consolidates the selection results of different variables using various approaches or principles to enhance the robustness of variable selection outcomes [21].

Stage 3 CKD can be divided into Stages 3a and 3b, representing mild and moderate renal function impairment, respectively. Both substages are vital in assessing whether a patient should undergo kidney dialysis, and they are associated with different mortality risks and clinical features [22,23]. However, only a few studies have explored how metabolic syndrome (MetS) affects Stage 3a and 3b CKD in patients and their shared risk factors [17]. While clinical health data, in general, can accumulate into a substantial amount of big data, in practice, whether addressing preventive healthcare for chronic diseases, including MetS, or assisting in the assessment of CKD at stages 3a-3b for high-risk diagnosis, establishing a risk prediction model for clinical use requires the incorporation of more appropriate or rational limited longitudinal healthcare data into research analysis. Collecting such data is essential for building predictive models and thereby identifying relevant risk factors more accurately, facilitating specialized physicians in clinical diagnosis, and aiding in medical decision making.

When dealing with limited medical datasets that are small or medium-sized, establishing conditional data under various variable ensemble rules can be beneficial in model building. ML algorithms, when applied with various variable ensemble rules, can compensate for the limitation of a small sample size, achieving a simultaneous improvement in predictive capabilities. Existing CKD analyses are primarily based on the findings of cross-sectional studies [24,25], which have mainly discussed CKD and evaluated its risk factors. The analysis of health examination data requires consideration of trends in the continuous change of data. That is, when conducting longitudinal data analysis, it is essential to give precedence to scrutinizing the trend and variability of the data, rather than exclusively depending on baseline data. Therefore, we generated four extended variables to gather trend and variability information from each of the predictor variables. These extended variables can provide a wide range of information and can be used as predictor variables for constructing the ML prediction model.

Given the significance of MetS as a risk factor for CKD and its pertinent role in CKD development mechanisms over times, evaluating the risk factors for CKD in patients with MetS using longitudinal data is a vital step in effectively managing and preventing CKD. This study aimed to use ML methods and VER schemes to identify the important risk factors for CKD in longitudinal data for patients with MetS diagnosed with stages 3a or 3b CKD. It assesses six effective ML methods—random forest (RF), multivariate adaptive regression splines (MARS), least absolute shrinkage and selection operator (Lasso), extreme gradient boosting (XGBoost), gradient boosting with categorical features support (CatBoost), and light gradient boosting machine (LightGBM)—as they are already being successfully utilized in various healthcare and medical applications [26,27,28,29], and five VERs in feature engineering—maximum aggregation (MA), arithmetic mean aggregation (AMA), geometric mean aggregation (GMA), Borda count aggregation (BCA), and ranking mean aggregation (RMA). Using these methods, we develop an ML- and VER-based hybrid multiphase CKD prediction scheme for evaluating and consolidating the key risk factors for patients with MetS and CKD. 

The proposed scheme first aggregates the scoring generated from corresponding ML methods via the five VERs. Because each machine learning model can provide feature importance scores on both numerical and categorical scales, the corresponding Variable Explanation Ratio (VER) is chosen based on these scores, allowing for the consideration of a wider range of information. Then, a union operation is employed to create a final selection of the most important features. By implementing the proposed scheme, we can reduce the complexity associated with a large number of features, thereby providing clinicians with crucial information to support medical decision making. Furthermore, as the proposed scheme can select important features, model performance can be improved when using these selected features. 

The accumulation of clinical health data often leads to big data challenges. However, creating effective risk prediction models for chronic diseases, such as MetS and high-risk CKD stages 3a-3b requires, focused longitudinal healthcare data analysis, which is often limited to small datasets. The limited longitudinal healthcare data make it challenging to build effective models in health promotion fields. This study innovates by proposing an effective scheme to enhance predictive accuracy with traditional machine learning algorithms, even with smaller datasets. The proposed scheme can effectively find features influencing CKD progression while improving the performance of the model in classifying CKD progression transitioning from stage 3a to 3b.

## 2. Materials and Methods

### 2.1. Data

This study used the regular health examination records of 71,108 patients in the Mei Jhao (MJ) Health Checkup-Based Population Database (MJPD), a Taiwanese long-term and continuous patient follow-up database, from 2005 to 2017. This timeframe was chosen to accumulate a sufficient number of consecutive samples. This decision was driven by the focus on a high-risk population with both CKD stages 3a to 3b and MetS. They included 201,807 health examination indicators and questionnaire records. We identified patients with MetS and stage 3a or 3b CKD using the MetS definition of the Health Promotion Administration (HPA) of the Ministry of Health and Welfare of Taiwan [30] and the KDIGO guidelines and references. This study was approved by the Institutional Review Board of the Far Eastern Memorial Hospital (approval number: 110027-E; approval date: 3 March 2021) and the MJ Health Research Foundation (authorization code: MJHRF2023004A) and was registered at ClinicalTrials.gov ID:NCT05225454, https://beta.clinicaltrials.gov/study/NCT05225454 (accessed on 27 February 2024).

### 2.2. Definitions of the Longitudinal Variables and Subjects

**Figure 1:**
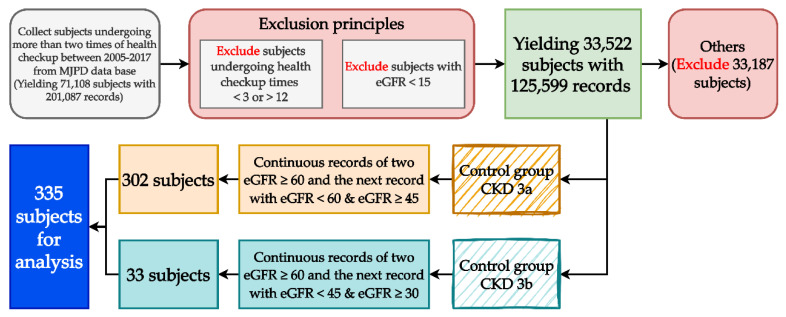
The process of identifying the longitudinal subjects.

This study constructed the longitudinal variables and their data by using each subject’s first two examination results to predict their third CKD examination results. We collected subjects’ 12-year examination records (from 2005 to 2017). Consistent with the prediction goals and conversion principles of the longitudinal data (each subject completed one examination annually), we excluded subjects with less than 3 or more than 12 examination records, leaving 33,533 subjects (125,641 health examination records). The data may include patients on dialysis, and they were not within the scope of this study, so we excluded 11 subjects whose estimated glomerular filtration rate (eGFR) was below 15 (42 records in total), leaving 33,522 subjects (125,599 records). We grouped these subjects based on the definition and eGFR criterion of CKD into an experimental group, a control group, and an “others” group. The experimental group comprised subjects with two consecutive eGFR values ≥60 in their health examination records; in total, 33 subjects met the definition of stage 3a CKD as their eGFR was ≥30 and <45. The control group comprised 302 subjects who met the definition of stage 3b CKD. The remaining 33,187 subjects were placed in the others group as they did not meet the criteria for the experimental or control group. After a multiphase processing of all subjects’ data, there were 335 eligible subjects. The process of identifying the longitudinal subjects is shown in Figure 1.

Among all 335 subjects, for each one, a total of three records were collected. As the aim of this study was to predict the relationship between each subject’s third CKD examination result and their risk factors, each subject’s previous two examination results were used as longitudinal predictor variables. Table 1 provides detailed descriptions and definitions of the predictor and target variables in the longitudinal data. The predictor variable $V_{i,t}$ represents the result of the ith variable at the tth examination (for example, the variable $V_{1,1}$ is the BF value at the first examination), and the objective variable Y represents the CKD result at the third examination. This study used 19 risk factors as predictor variables and can be further defined as Equation (1).

(1) $$V_{i,t},\;\forall i,\;t\in N\;\mathrm{where}\;i=1,2,\ldots ,19;t=1,2.$$

To generate extended variables, the four statistics involving the closest value, mean value, standard deviation (SD) value, and difference value of a predictor variable are considered. The closest value of a predictor variable uses the subjects’ latest examination results, which is the second examination in this study ($V_{i,2}$). The predictor variable ($V_{i}C$) generated based on the closest value is defined as Equation (2). For example, $V_{1}C$ is the BF record ($V_{1,2}$) at the second examination, and it can be abbreviated as BF(C).The predictor variable ($V_{i}M$) generated using the mean value of a predictor variable is the mean of the previous two examination results $\left(V_{i,1},V_{i,2}\right)$, and it can be defined as Equation (3). For example, $V_{1}M$ is generated by obtaining the mean BF value of the last two examinations, and it can be abbreviated as BF(M). The predictor variable ($V_{i}S$) is the SD of the last two examination results$\;\left(V_{i,1},V_{i,2}\right)$ of a predictor variable, and it can be defined as Equation (4). For example, $V_{1}S$ is generated by obtaining the SD of the BF result ($V_{1}S)\;$at the first and second examinations. $V_{1}S$ can be abbreviated as BF(S). A predictor variable ($V_{i}D$) is the difference between the last two examination results$\;\left(V_{i,1},V_{i,2}\right)$ of a predictor variable, and it can be defined as Equation (5). For example, $V_{1}D\;$is generated by subtracting the BF results of the first and second examinations. $V_{1}D$ can be abbreviated as BF(D).

All four of the statistical approaches to generating extended variables are applied to all 19 predictor variables to generate the predictor variables for analysis. Therefore, a total of 76 predictor variables are considered, and they are also used to construct the ML prediction model. The demographics of the 19 variables from the subjects’ latest examination ($V_{i}C$) are shown in Table S1 in the Supplementary Materials.

(2) $$V_{i}C=V_{i,2}$$

(3) $$V_{i}M=\frac{V_{i,1}+V_{i,2}}{2}$$

(4) $$V_{i}S=\sqrt{\frac{{\left(V_{i,1}-V_{i}M\right)}^{2}+{\left(V_{i,2}-V_{i}M\right)}^{2}}{2-1}}$$

(5) $$V_{i}D=V_{i,2}-V_{i,1}$$

### 2.3. Proposed Multiphase Hybrid Risk Factor Evaluation Scheme

In order to predict CKD outcomes and identify the key risk factors for CKD, this study proposes a multiphase hybrid CKD prediction scheme grounded in six ML algorithms (RF, MARS, Lasso, XGBoost, CatBoost, and LightGBM) that utilize the longitudinal variables generated in the previous section. RF is an ensemble learning method that consists of decision trees combined by bagging (bootstrap aggregation) [31]. MARS is a multivariate, nonlinear, nonparametric regression method combining recursive partitioning and piecewise polynomial functions [32]. Lasso shrinks predictor variables with weaker contributions to zero to control the trade-off between the bias and the variance in model fitting while reducing the likelihood of overfitting [33]. 

XGBoost is an ensemble learning method based on gradient boosting [34]. CatBoost is an improved decision tree algorithm that combines ordered boosting, gradient boosting, and classification features [35]. LightGBM is a histogram-based distributed gradient boosting framework algorithm that restricts the maximum depth of the decision trees [36]. These ML methods have been used successfully in various healthcare and medical applications [26,27,28,29], and all of them have the ability to select features while providing importance scoring to the input features. To evaluate the performance of the ML models, the balanced accuracy (BA), sensitivity (SEN), specificity (SPE), and area under the receiver operating characteristic (ROC) curve (AUC) are used. Furthermore, as it is widely utilized in many clinical-related studies, logistic regression (LGR) is also considered as the benchmark in this study to ensure all six of the ML models have reasonable performance. 

**Figure 2:**
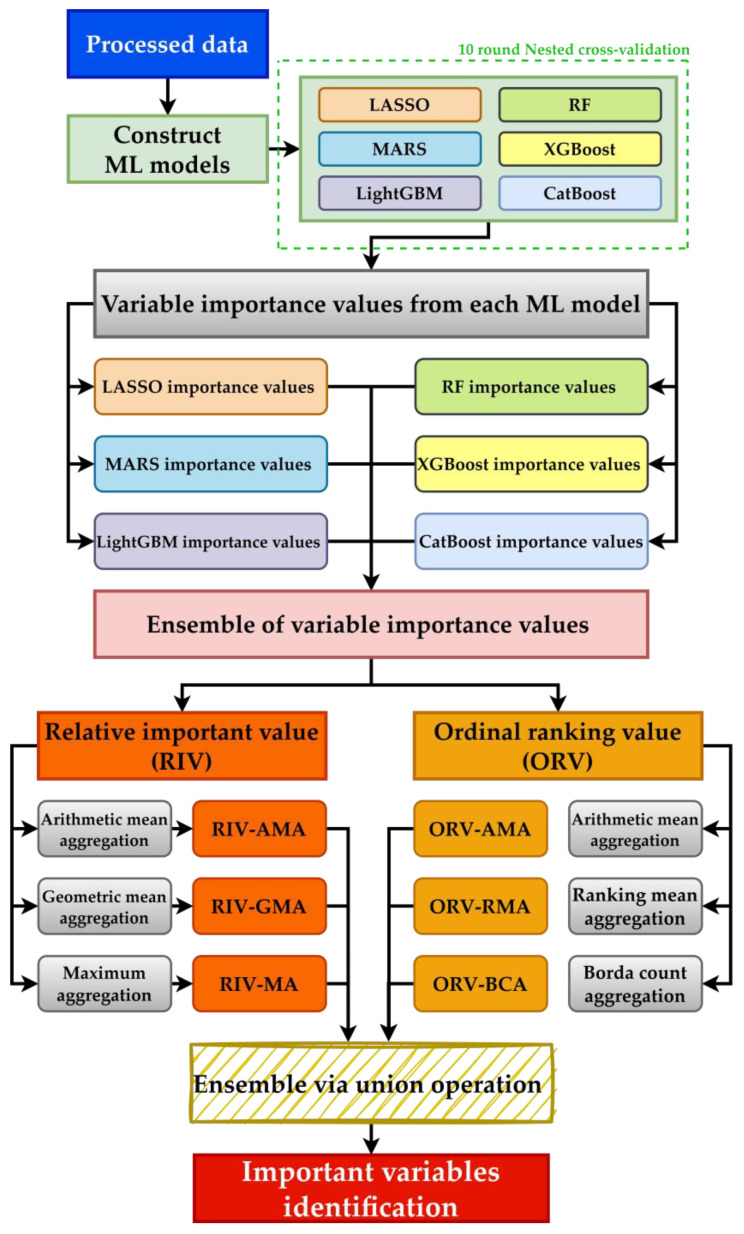
Proposed multiphase hybrid risk factor evaluation scheme.

The procedure of the hybrid multiphase CKD prediction scheme is shown in Figure 2. As shown in the figure, after obtaining the data with the generated longitudinal variables, ML models are constructed using the data. Additionally, an oversampling technique is utilized to address the class imbalance issue in the data. With the built ML models, the relevant importance value of each variable can be extracted from each ML model. Because each model has different hyper-parameters required to be tuned, 10-fold nested cross-validation (10f-NCV) is utilized for hyper-parameter tuning. Under the structure of 10f-NCV, in one iteration, the data are randomly split into 10 folds, where 1 fold is used for testing and the rest of the 9 folds are used for testing. During training, the 9 folds of the data will be further split into 8 folds for training and 1 fold for validation. Training ends when all of the 9 folds of data are used for validation once and the optimized hyper-parameter sets are found; then, the testing fold is used for evaluation. The entire 10f-NCV process is finished when all folds are used for testing once (a total of 10 iterations). 

After constructing valid ML models, each can generate relevant information for each variable according to its model rules, thus generating two variable importance values: the ratio-scale-based relative importance value (RIV) and the ordinal-scale-based ordinal ranking value (ORV). In the RIV, the values of the most and least important variables are 100 and 0, respectively. The RIV is ordered from highest to lowest, and the given ranking value is the ORV. The most important variable, whose RIV is the highest or equal to 100, is placed at the top; the least important variable, whose RIV is the lowest or equal to 0, is placed at the bottom. Values can be repeated, which means that the variable importance of two or more variables may be similar. As each optimal ML method was repeated 10 times, there will be 10 corresponding variable importance values that are distinctive to each method. Each method’s mean importance was calculated to yield its single merged RIV and ORV.

Because a single selection variable algorithm has the propensity to choose a locally optimal solution, the ensemble variable has more opportunities to better approximate the optimal solution by averaging different assumptions [37]. To derive more stable results, different VER approaches are considered. VER approaches can provide more robust variable selection results than a single variable selection method and reduce bias and variance. It has shown excellent results across various research domains [38,39]. Hence, MA, AMA, GMA, BCA, and RMA are used in this study as they are widely utilized in many studies [37,40]. Moreover, because different VER approaches are only applicable to a specific variable measurement scale, the RIV variable integration is based on MA, AMA, and GMA, whereas ORV is based on AMA, BCA, and RMA. The equations of each VER approach used are as follows:

(6) $$AMA_{F_{i}}=\frac{1}{j}\overset{j}{\underset{k=1}{\sum }}r_{ik}=\frac{1}{j}\left(r_{i1}+r_{i2}+\cdots +r_{ij}\right)$$

(7) $$GMA_{F_{i}}={\left(\overset{j}{\underset{k=1}{\prod }}r_{ik}\right)}^{\frac{1}{j}}=\sqrt[{j}]{r_{i1}r_{i2}\ldots r_{ij}}$$

(8) $$MA_{F_{i}}=Max\left(r_{i1},r_{i2},\ldots ,r_{ij}\right)$$

(9) $$RMA_{F_{i}}=Median\left(r_{i1},r_{i2},\ldots ,r_{ij}\right)$$

(10) $$BCA_{F_{i}}=\mathrm{Mode}\left(Count\left(r_{i1},r_{i2},\ldots ,r_{ij}\right)\right)$$

where $r_{ij}$ is the RIV or ORV of the ith variable in the jth method. After aggregation via VER approaches, six sets of variable importance rankings are generated, namely RIV-AMA, RIV-GMA, RIV-MA, ORV-AMA, ORV-RMA, and ORV-BCA. Finally, union operation is used to integrate and compare the six importance ranking sets and to identify the most important risk factors for discussion.

This study used the R programming language (version 4.0.5; http://www.R-project.org (accessed on 27 February 2024)) and RStudio software (version 1.1.453; https://www.rstudio.com/products/rstudio/ (accessed on 27 February 2024)) to construct an effective ML model. All of the algorithm equations and estimated optimal hyperparameters of the models were built using R-related software packages. The package information is as following: The RF, LGR, MARS, Lasso, XGBoost, CatBoost, and LightGBM models were created using the randomForest (version 4.7-1.1) [41], stats (version 4.0.5), earth (version 5.3.1) [42], glmnet (version 4.1-7) [43], xgboost (version 1.6.0.1) [44], catboost (version 0.25.1) [45], and lightgbm (version 3.3.2) [46] packages, respectively. Lastly, the optimal hyperparameters were estimated for all models using the caret package (version 6.0-93) [47].

## 3. Results

**Figure 3:**
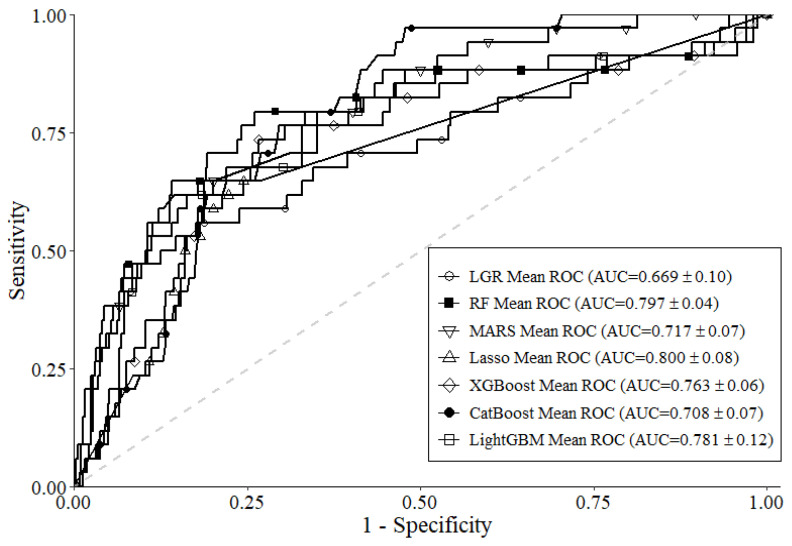
ROC curves of each ML model.

Table 2 shows the average performance of each ML model after 10f-NCV. As shown in the table, Lasso had the best BA of 0.813, LightGBM had the best SEN of 0.791, and RF had the best SPE of 0.898. Lasso had the best AUC of 0.800, and all six ML models had greater scores of AUC than the benchmark LGR model (AUC 0.669). This can also be found in the ROC curve presented in Figure 3. Overall, according to the results in Table 2 and Figure 3, the usage of all six ML models for ensemble to identify important variables is reasonable.

The variable importance value of each variable generated from each ML model in terms of RIV (Table 3) and ORV (Table 4) can be found in the tables. In Table 3, the first 12 variables are presented. As different ML models analyze the data with different approaches and mechanisms, it can be seen that each ML model yields a different RIV for each variable. For example, both Lasso and LightGBM yield the lowest RIV of zero to $V_{1}\left(S\right)$, whereas the other four ML methods yield a relatively higher RIV. The same concept can be found in Table 4. For example, $V_{3}\left(M\right)$ is ranked relatively lower by MARS (ORV 26) than the other five ML methods. Next, in order to derive more stable results and considerations when identifying important variables, the results of RIV and ORV are aggregated via the VER approaches, which are presented in Table 5.

Table 5 presents the first 12 variables’ aggregation results from RIVs and ORVs via different VER approaches. As the characteristic of RIV, the aggregated importance values are generated from the six ML models with the corresponding VER equations. The aggregated importance value ranges between 0 and 100, and more important variables will have higher values. This concept suits all of the aggregated RIVs (RIV-AMA, RIV-GMA, and RIV-MA). Both the aggregations of ORV-AMA and ORV-RMA have similar concepts as RIVs, but with slight differences due to the characteristics of ORV. As the most important variable will be assigned the rank of one under the structure of ORV, the more important variable will have a value closer to one after aggregation. Under ORV-BCA, when two or more ML models are assigned the same ranking to a variable, that specific ranking will be the aggregated value of the variable. Taking ORVs of $V_{1}\left(C\right)$ in Table 4 as an example, because both MARS and Lasso assigned the ranking of 70 to $V_{1}\left(C\right)$, the aggregated value of $V_{1}\left(C\right)$ in ORV-BCA is 70, and this can also be seen in Table 5. Furthermore, if all six ML models have assigned separate rankings to a variable, the worst-case scenario will be taken into consideration, in which the aggregated value will be the worst-ranking value. For example, $V_{3}\left(M\right)$ in Table 4 can be found with separated rankings assigned from each ML model. Because the worst ranking of $V_{3}\left(M\right)$ is 26, the ORV-BCA of $V_{3}\left(M\right)$ is 26. Overall, as shown in Table 5, with VER approaches, different information regarding the variables analyzed by each ML method can be brought into consideration. To better compare and interpret the rankings of each variable via VER approaches, the results in Table 5 can be further organized into Table 6.

Table 6 presents the top 12 ranking variables of RIV and ORV based on the corresponding VER approaches. As shown in the table, BUN(S) is the most important variable across all six aggregation results utilizing VER approaches; BUN(M) is the second most important variable, which only ranked the third in RIV-MA. Overall, the top three ranking important variables are similar and begin to vary in lower rankings.

To examine the association between important variables using different VER approaches, union operation is performed on Table 6, and the results are organized into Table 7. As presented in the table, important variables identified after union operation in different ranking combinations can be seen. Five conditions of the combination for union operation are used, which are within the first 4 rankings, within the first 6 rankings, within the first 8 rankings, within the first 10 rankings, and within the first 12 rankings. Taking the first condition (within the first four rankings) for the RIV rule as an example, the first four ranking variables from each aggregation rule in Table 6 have to be identified first, then, with union operation, BUN(S), BUN(M), Hb(S), RBC(S), and r-GT(M) can be found, thus satisfying the condition in Table 7. The same process is performed for all of the other conditions for both rules.

To evaluate the stability of the union operation results of the selected variable sets under the two proposed aggregation rules, Lasso is constructed based on variables selected in each variable combination condition of RIV and ORV from Table 7, as the preliminary ML model performance results revealed that Lasso is the best one in this study. The performance of Lasso with different variable combination condition sets is shown in Table 8. According to the table, all AUCs of the union operations of the ranked variables under the two rules were greater than 0.804. Notably, variables within the first eight ranking conditions of RIV yield the best AUC of 0.883. Lasso uses 13 variables in total under the best variable combination condition; on the other hand, Lasso using all 76 variables has lower performance, with an AUC of 0.800. Therefore, the results are improved after variable selection, which greatly improves the overall prediction performance.

**Figure 4:**
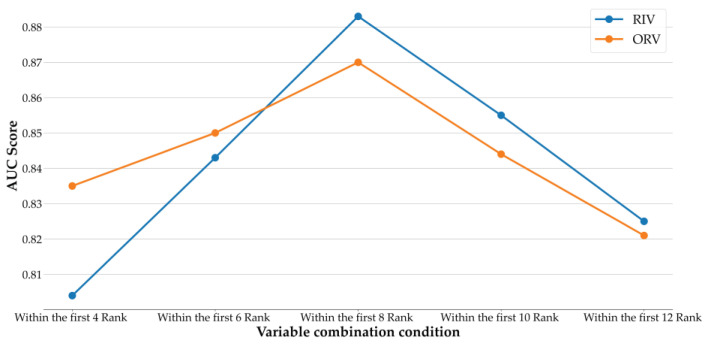
AUC of Lasso with different variable combination conditions.

Figure 4 shows the AUC values of Lasso using different variable combination conditions with RIV and ORV. As shown in the figure, both RIV and ORV have increasing AUC values from the condition within the first four rankings to within the first eight rankings, and then both of their AUCs begin to decrease. ORV has better performance in AUC than RIV when the conditions are within the first four rankings and within the first six rankings; after that, RIV is superior to ORV in AUC when the amount of variables increases. In summary, the stability of the union operation is confirmed, and it indicates that the proposed risk factor evaluation scheme of this study can provide promising results.

## 4. Discussion

While clinical health data, in general, can accumulate into a substantial amount of big data, in practice, whether addressing preventive healthcare for chronic diseases, including metabolic syndrome (MetS), or assisting in the assessment of chronic kidney disease (CKD) at stages 3a-3b for high-risk diagnosis, the establishment of a risk prediction model for clinical use requires more appropriate or rational limited longitudinal healthcare data for research analysis. Collecting such data is essential to build predictive models, thereby identifying relevant risk factors more accurately, facilitating specialized physicians in clinical diagnosis, and aiding in medical decision making.

The analysis of clinical data often faces the challenge of dealing with limited sample sizes and complex variable interactions. Employing methods that consider multiple aspects can help compensate for these limitations. As the information can vary from different ML methods, consideration of VERs and union operation to aggregate them could enhance the overall predictive capability. Moreover, aggregated information, such as important features, can support healthcare or actual clinical scenarios. Furthermore, the analysis of health examination data requires consideration of the trends in the continuous change of data. Analyzing longitudinal data that meet the conditions is of greater research value and contributes to the subsequent predictive benefits of this study. Predicting CKD progression risk is a vital task in clinical management. 

CKD is a progressive kidney disease characterized by deteriorating renal function. ML methods have been successfully used to predict CKD risk. This study used ML methods and feature engineering to construct a longitudinal variable set scheme to identify patients with MetS diagnosed with stages 3a or 3b CKD. Its results are highly significant for patients. For example, early CKD detection is conducive to providing effective interventions and measures to high-risk patients. Early treatment often leads to favorable treatment outcomes in the disease course. Next, in our longitudinal analysis, all six ML methods outperformed conventional LGR, and the Lasso model was the best, as reflected by its AUC of 0.800, which was 2.8% and 13.1% higher than that of conventional LGR. Our findings corroborate those of previous studies. Indeed, ML methods, especially Lasso [48], can be used to resolve the classification bias in several categories while showing strong prediction performance with imbalanced data [18].

In addition, this study used variable ensemble and union operation to integrate the ML-selected variable results and analyzed the Lasso-selected variables. Its experimental results demonstrated that the variable ensemble methods and union operation all effectively improved the Lasso model’s prediction performance. We offer reliable estimations of CKD risk factors based on different VERs. Specifically, we reduced the number of cross-sectional variables from 76 to 13 through variable selection, enhancing prediction performance. As reflected by the AUC, prediction performance increased from 5.6% to 8.3% after variable selection with Lasso. Like previous studies, the proposed hybrid scheme outperformed standalone schemes, as variable selection identified the important CKD variables and increased the model’s prediction performance [49,50,51]. The results with the selected variables were similar to those of existing clinical studies. For example, Lasso identified BUN, Hb, RBC, r-GT, HDL, UA, SBP, and FPG as key risk factors for CKD based on the cross-sectional results. The associations between these risk factors and CKD are elaborated based on previous studies. 

Past related publications have not emphasized the analysis of longitudinal data in small to medium-sized samples. For health or clinical data with two or more real instances, the consideration of statistics like closest (C), mean (M), standard deviation (S), and difference (D) among variables has been lacking. In this study, not only did we identify the significance of these variables (Hb, RBC, BUN, r-GT, HDL, UA, SBP, and FPG) again, but we also delved deeper to explore which statistical values among these continuous data variables hold more meaning. Hb or RBCs are important indicators of the blood’s oxygen-carrying capacity. An excessively low value can lead to anemia, a common complication of CKD and a recognized risk factor for CKD deterioration [52,53]. A survey found that 41% of 209,311 patients with CKD had anemia [54]. Accordingly, study results found that in longitudinal data, the two variable statistical patterns, Hb(S), RBCs(M), and RBC(S), possess greater predictive capabilities. 

BUN is an independent risk factor for CKD [27]. Increasing studies have examined the relationship between CKD and BUN. For example, BUN and CKD are positively correlated. Moreover, Seki et al. (2019) also reported that BUN may predict kidney disease development [55]. This study found that in longitudinal data, the important variable BUN may be more meaningfully assessed through the BUN(C), BUN(D), BUN(S), and BUN(M) values of each examination. Many studies have stressed the importance of UA in CKD. One of those studies identified the UA level as an important predictor of CKD [56]. A recent study observed that higher UA levels correlated significantly with CKD in middle-aged men regardless of their BMI [57]. Based on the findings of this study, it was discovered that in longitudinal data, the important variable UA may be more meaningfully assessed through the standard deviation “UA(S)” values of its multiple examinations.

r-GT is an enzyme found on the cell surface of all tissues. It is a typical indicator of alcohol consumption and hepatic impairment. Increasing studies have identified r-GT as an independent risk factor for CKD and ESRD [58]. For example, two Japanese studies concurred that increased r-GT correlates positively with CKD [59,60]. A study on male South Korean workers found that r-GT and CKD correlated positively, and r-GT may predict early CKD [61]. The research results indicate that exploring the mean value r-GT(M) of the variable r-GT(D) over longitudinal data may be more meaningful.

A recent 7472 person-years follow-up study in Korea showed that in patients with CKD, higher SBP and DBP levels were associated with a higher risk of a composite kidney outcome reflecting CKD progression. SBP had a greater association with adverse kidney outcomes than DBP [62]. Blood pressure control is undoubtedly an important risk factor for CKD, but for the CKD high-risk group during 3a to 3b, the variability of systolic blood pressure DBP(S) may be cause for concern. There exists strong evidence that HDL is associated with patients with impairment of kidney function and/or progression of CKD. HDL-C concentrations, the composition of HDL particles, disturbances in functionality, and especially the reverse cholesterol transport, might be different between various stages of kidney impairment, especially between patients with and without nephrotic syndrome [63]. Furthermore, a Mendelian randomization study showed HDL-C, LDL-C, and Triglycerides as CKD risk factors [64]. Therefore, the variance of HDL(S) may cause concern for CKD during 3a to 3b.

To summarize, blood sugar management is conducive to preventing diabetes, nephropathy, and other diabetic microvascular complications. Research has identified FPG as an important risk factor for CKD [52]. A retrospective study by Cao et al. (2022) [65] identified age, sex, BMI, T2DM, FPG, stroke, and hypertension as risk factors for CKD. Regarding the FPG values, the results of this study indicate that investigating the difference in “FPG(D)” from the previous test as a research variable may have a higher predictive value for risk assessment.

## 5. Limitations and Future Recommendations

While this study utilized innovative applications and a set of ensemble variable analyses for continuous data in small and medium-sized health data samples, clinical validation requires consideration of regional and ethnic differences. These variations may affect the construction of models and lead to differences in research outcomes across different regions and ethnic groups in subsequent studies. Due to the limitation of the research dataset, not every subject has completed, multi-year data, so only the most recent three data points from the longitudinal data are selected. Additionally, the proposed scheme is restricted to the ML methods’ mechanism having the ability to provide feature importance scoring. Some methods, such as neural network or k-nearest neighbor, may not be applicable to our scheme, as their algorithm design cannot provide feature importance scoring. Future research can consider more points of data analysis. Avoiding the biases in data collection or model assumptions would strengthen the validity of the findings. Exploring the potential for future research directions, such as validation of the predictive model in a clinical setting or investigating the impact of early intervention based on the identified risk factors, could add depth to the study’s implications. Regarding concerns about ethnic and regional differences and recommendations from clinical guidelines, follow-up research can apply the prediction model of this study to other healthcare settings or patient populations to improve scalability and generalizability; at the same time, based on the identified risk factors discovered in this study, an evaluation of the impact of early intervention could be performed.

## 6. Conclusions

The analysis of health screening data requires consideration of the trends in the continuous change of data. Analyzing longitudinal data that meet the conditions is of greater research value and contributes to the subsequent predictive benefits of this study. The hybrid multiphase scheme for predicting CKD in patients with MetS developed in this study through ML methods and feature engineering showed strong prediction performance. The limited longitudinal health screening data based on different feature ensembles demonstrated that the hybrid multiphase scheme effectively improved ML predictive performance. This study also examined common risk factors affecting CKD in patients with MetS using different models and ranked their importance for future reference. These rankings not only facilitate kidney condition assessment based on the risk factors but also the detection of other underlying diseases that patients with CKD might have. Moreover, our results are generalizable to a certain extent and may be used to enhance the understanding and treatment of other diseases by using the same ML methods and similar hybrid schemes. For healthcare professionals, information on how to incorporate the findings of this study into their clinical practice or decision-making processes would be beneficial. Based on the analysis results of this case study, for preventing CKD in the sub-healthy population with MetS, it is predictable that well-known factors like BUN, UA, and PFG play crucial roles. However, lesser-discussed factors, such as Hb, RBCs, or r-GT, should receive more attention in clinical practice or decision-making processes, which could be beneficial. Additionally, observing the mean, standard deviation, and difference of different variables across three consecutive data points carries distinct implications. The study’s findings may inform personalized medicine and targeted interventions for potential patients with high-risk CKD, thereby identifying clinical implications of risk factors and real-world healthcare applications. Validating the prediction model in a clinical setting and externally aligns with existing guidelines, potentially enhancing current CKD management practices.

## Figures and Tables

**Table 1:** Definitions and descriptions of the predictor and target variables.

	Variable	Description	Unit
$V_{1,t}$	Body Fat (BF)	BF of subject at tth examination	%
$V_{2,t}$	Body Mass Index (BMI)	BMI of subject at tth examination	kg/m^2^
$V_{3,t}$	Blood Urea Nitrogen (BUN)	BUN of subject at tth examination	mg/dL
$V_{4,t}$	Diastolic Blood Pressure (DBP)	DBP of subject at tth examination	mmHg
$V_{5,t}$	Fasting Plasma Glucose (FPG)	FPG of subject at tth examination	mg/dL
$V_{6,t}$	Hemoglobin (Hb)	Hb of subject at tth examination	g/dL
$V_{7,t}$	Hip Circumference (HC)	HC of subject at tth examination	cm
$V_{8,t}$	High-Density Lipoprotein Cholesterol (HDL)	HDL of subject at tth examination	mg/dL
$V_{9,t}$	Intraocular Pressure (IOP)	IOP of subject at tth examination	mmHg
$V_{10,t}$	Low-Density Lipoprotein Cholesterol (LDL)	LDL of subject at tth examination	mg/dL
$V_{11,t}$	Mean Cell Volume (MCV)	MCV of subject at tth examination	fl
$V_{12,t}$	Red Blood Cells (RBCs)	RBCs of subject at tth examination	10^6^/μL
$V_{13,t}$	Gamma Glutamyl Transpeptidase (r-GT)	r-GT of subject at tth examination	U/L
$V_{14,t}$	Systolic Blood Pressure (SBP)	SBP of subject at tth examination	mmHg
$V_{15,t}$	Serum Glutamic Oxaloacetic Transaminase (SGOT)	SGOT of subject at tth examination	U/L
$V_{16,t}$	Serum Glutamic Pyruvic Transaminase (SGPT)	SGPT of subject at tth examination	U/L
$V_{17,t}$	Triglyceride (TG)	TG of subject at tth examination	mg/dL
$V_{18,t}$	Uric Acid (UA)	UA of subject at tth examination	mg/dL
$V_{19,t}$	Waist Circumference (WC)	WC of subject at tth examination	cm
Y	Chronic Kidney Disease (CKD)	CKD result of subject at the third examination	

**Table 2:** Average performance of each ML model after 10f-NCV.

Model	BA (SD)	SEN (SD)	SPE (SD)	AUC (SD)
LGR	0.704 (0.09)	0.656 (0.32)	0.752 (0.19)	0.669 (0.10)
RF	0.798 (0.04)	0.699 (0.12)	0.898 (0.15)	0.797 (0.04)
MARS	0.766 (0.04)	0.752 (0.09)	0.780 (0.12)	0.717 (0.07)
Lasso	0.813 (0.05)	0.769 (0.06)	0.856 (0.12)	0.800 (0.08)
XGBoost	0.769 (0.05)	0.741 (0.13)	0.797 (0.18)	0.763 (0.06)
CatBoost	0.763 (0.06)	0.777 (0.16)	0.750 (0.14)	0.708 (0.07)
LightGBM	0.780 (0.10)	0.791 (0.15)	0.770 (0.17)	0.781 (0.12)

**Table 3:** First 12 RIVs of each variable from the six used ML models.

Vars	RF	MARS	Lasso	XGBoost	CatBoost	LightGBM
$V_{1}\left(C\right)$	24.00	1.41	0.22	9.22	10.43	3.78
$V_{1}\left(M\right)$	23.37	13.20	0.00	9.30	14.07	0.43
$V_{1}\left(S\right)$	9.36	1.12	0.00	2.88	12.38	0.00
$V_{1}\left(D\right)$	12.09	6.95	0.00	10.31	17.16	1.40
$V_{2}\left(C\right)$	10.92	0.00	0.00	1.92	2.34	0.02
$V_{2}\left(M\right)$	8.10	3.70	0.00	1.08	8.57	0.11
$V_{2}\left(S\right)$	9.58	8.35	0.00	1.81	16.79	0.12
$V_{2}\left(D\right)$	15.97	0.00	0.00	8.56	10.91	0.31
$V_{3}\left(C\right)$	22.97	24.22	0.00	3.04	17.98	2.38
$V_{3}\left(M\right)$	56.80	48.17	10.73	40.22	46.50	11.82
$V_{3}\left(S\right)$	99.03	100.00	53.95	100.00	94.22	100.00
$V_{3}\left(D\right)$	17.44	31.61	0.00	4.85	6.83	0.66
…	…	…	…	…	…	…

**Table 4:** First 12 ORVs of each variable from the six used ML models.

Vars	RF	MARS	Lasso	XGBoost	CatBoost	LightGBM
$V_{1}\left(C\right)$	18	70	70	25	39	34
$V_{1}\left(M\right)$	23	62	76	16	34	42
$V_{1}\left(S\right)$	51	70	76	46	41	71
$V_{1}\left(D\right)$	37	64	76	18	41	38
$V_{2}\left(C\right)$	43	76	76	50	62	67
$V_{2}\left(M\right)$	57	70	76	61	48	66
$V_{2}\left(S\right)$	44	64	76	53	21	64
$V_{2}\left(D\right)$	29	76	76	25	40	60
$V_{3}\left(C\right)$	10	37	76	48	34	50
$V_{3}\left(M\right)$	2	26	4	3	6	11
$V_{3}\left(S\right)$	2	1	3	1	2	1
$V_{3}\left(D\right)$	21	36	76	32	43	47
…	…	…	…	…	…	…

**Table 5:** First 12 aggregated RIVs and ORVs via VER approaches of each variable.

Vars	RIV-AMA	RIV-GMA	RIV-MA	ORV-AMA	ORV-RMA	ORV-BCA
$V_{1}\left(C\right)$	8.18	3.73	24.00	42.67	36.50	70
$V_{1}\left(M\right)$	10.06	0.00	23.37	42.17	38.00	76
$V_{1}\left(S\right)$	4.29	0.00	12.38	59.17	60.50	76
$V_{1}\left(D\right)$	7.98	0.00	17.16	45.67	39.50	76
$V_{2}\left(C\right)$	2.53	0.00	10.92	62.33	64.50	76
$V_{2}\left(M\right)$	3.59	0.00	8.57	63.00	63.50	76
$V_{2}\left(S\right)$	6.11	0.00	16.79	53.67	58.50	76
$V_{2}\left(D\right)$	5.96	0.00	15.97	51.00	50.00	76
$V_{3}\left(C\right)$	11.76	0.00	24.22	42.50	42.50	76
$V_{3}\left(M\right)$	35.71	29.42	56.80	8.67	5.00	26
$V_{3}\left(S\right)$	91.20	89.19	100.00	1.67	1.50	1
$V_{3}\left(D\right)$	10.23	0.00	31.61	42.50	39.50	76
…	…	…	…	…	…	…

**Table 6:** Top 12 ranking variables of RIV and ORV with different VER approaches.

	RIV			ORV		
Rule/Rank	RIV-AMA	RIV-GMA	RIV-MA	ORV-AMA	ORV-RMA	ORV-BCA
1	BUN(S)	BUN(S)	BUN(S)	BUN(S)	BUN(S)	BUN(S)
2	BUN(M)	BUN(M)	Hb(S)	BUN(M)	BUN(M)	BUN(M)
3	Hb(S)	Hb(S)	BUN(M)	Hb(S)	Hb(S)	LDL(M)
4	RBC(S)	RBC(S)	r-GT(M)	RBC(S)	FPG(D)	HDL(S)
5	r-GT(M)	RBC(M)	RBC(S)	FPG(D)	RBC(S)	TG(S)
6	HDL(S)	UA(S)	HDL(S)	HDL(S)	HDL(S)	HC(S)
7	BUN(C)	SBP(S)	r-GT(D)	RBC(M)	RBC(M)	UA(S)
8	RBC(M)	FPG(D)	BUN(D)	SBP(S)	Hb(M)	BMI(S)
9	r-GT(D)	BF(C)	RBC(M)	r-GT(M)	SBP(M)	DBP(D)
10	LDL(D)	SBP(C)	WC(C)	SBP(M)	BF(C)	Hb(M)
11	FPG(D)	Hb(M)	LDL(D)	Hb(M)	UA(S)	SGOT(D)
12	BUN(D)	DBP(S)	RBC(C)	BF(M)	BF(M)	BF(C)

**Table 7:** Important Variables identified after union operation in different ranking combinations.

Rules	Variable Combination Conditions	Selected Important Variables after Union Operation
RIV	Within the first 4 rankings	BUN(S), BUN(M), Hb(S), RBC(S), r-GT(M)
	Within the first 6 rankings	BUN(S), BUN(M), Hb(S), RBC(S), r-GT(M), RBC(M), HDL(S), UA(S)
	Within the first 8 rankings	BUN(S), BUN(M), Hb(S), RBC(S), r-GT(M), RBC(M), HDL(S), UA(S), BUN(C), SBP(S), r-GT(D), FPG(D), BUN(D)
	Within the first 10 rankings	BUN(S), BUN(M), Hb(S), RBC(S), r-GT(M), RBC(M), HDL(S), UA(S), BUN(C), SBP(S), r-GT(D), FPG(D), BUN(D), BF(C), LDL(D), SBP(C), WC(C)
	Within the first 12 rankings	BUN(S), BUN(M), Hb(S), RBC(S), r-GT(M), RBC(M), HDL(S), UA(S), BUN(C), SBP(S), r-GT(D), FPG(D), BUN(D), BF(C), LDL(D), SBP(C), WC(C), Hb(M), DBP(S), RBC(C)
ORV	Within the first 4 rankings	BUN(S), BUN(M), Hb(S), LDL(M), RBC(S), FPG(D), HDL(S)
	Within the first 6 rankings	BUN(S), BUN(M), Hb(S), LDL(M), RBC(S), FPG(D), HDL(S), TG(S), HC(S)
	Within the first 8 rankings	BUN(S), BUN(M), Hb(S), LDL(M), RBC(S), FPG(D), HDL(S), TG(S), HC(S), RBC(M), UA(S), SBP(S), Hb(M), BMI(S)
	Within the first 10 rankings	BUN(S), BUN(M), Hb(S), LDL(M), RBC(S), FPG(D), HDL(S), TG(S), HC(S), RBC(M), UA(S), SBP(S), Hb(M), BMI(S), r-GT(M), SBP(M), DBP(D), BF(C)
	Within the first 12 rankings	BUN(S), BUN(M), Hb(S), LDL(M), RBC(S), FPG(D), HDL(S), TG(S), HC(S), RBC(M), UA(S), SBP(S), Hb(M), BMI(S), r-GT(M), SBP(M), DBP(D), BF(C), SGOT(D), BF(M)

**Table 8:** AUC of Lasso with different variable combinations according to the results in Table 7.

Rule	Variable Combination Conditions (Number of the Selected Variable)	AUC
RIV	Within the first 4 rankings (5)	0.804
	Within the first 6 rankings (8)	0.843
	Within the first 8 rankings (13) *	0.883
	Within the first 10 rankings (17)	0.855
	Within the first 12 rankings (20)	0.825
ORV	Within the first 4 rankings (7)	0.835
	Within the first 6 rankings (9)	0.850
	Within the first 8 rankings (14)	0.870
	Within the first 10 rankings (20)	0.844
	Within the first 12 rankings (19)	0.821

* represents the best AUC value.

## Data Availability

The datasets generated during and/or analyzed during the current study are not publicly available due to ethical restrictions. The data acquisition process requires approval from the Institutional Review Board (IRB) and authorization from the MJ Health Research Foundation (MJHRF). For more details regarding the data application procedures, please refer to https://www.mjhrf.org/main/page/release1/en/#release01 (accessed on 27 February 2024).

## Supplementary Materials

The following supporting information can be downloaded at: https://www.mdpi.com/article/10.3390/diagnostics14080825/s1, Table S1: The demographics of the 19 variables from the subjects’ latest examination ($V_{i,}C$).
