# Supplementary material for: Analyzing Longitudinal Health Screening Data with Feature Ensemble and Machine Learning Techniques: Investigating Diagnostic Risk Factors of Metabolic Syndrome for Chronic Kidney Disease Stages 3a to 3b
Source: Diagnostics (Basel). 2024 Apr 17;14(8):825. doi: 10.3390/diagnostics14080825 (PMC11048899; doi:10.3390/diagnostics14080825)
Supplement: Supplementary file 1 [file diagnostics-14-00825-s001.zip › diagnostics-2913524-supplementary.pdf]

Supplement:

Table S1. The demographics of the 19 variables from the subjects' latest examination ($V_{i,}C$)

Variable	Mean(SD)	Variable	Mean(SD)
BF	30.32 ± 6.99	MCV	43.19 ± 4.34
BMI	27.19 ± 3.29	RBCs	4.84 ± 0.54
BUN	18.54 ± 4.74	r-GT	38.95 ± 45.14
DBP	84.30 ± 12.50	SBP	138.18 ± 20.54
FPG	123.20 ± 34.35	SGOT	28.61 ± 13.88
Hb	14.48 ± 1.47	SGPT	34.19 ± 21.16
HC	99.28 ± 6.22	TG	194.49 ± 78.31
HDL	47.36 ± 9.98	UA	7.43 ± 1.64
IOP	14.56 ± 3.34	WC	90.21 ± 8.50
LDL	123.30 ± 34.39		
